# Morphology Controlled Fabrication of InN Nanowires on Brass Substrates

**DOI:** 10.3390/nano6110195

**Published:** 2016-10-29

**Authors:** Huijie Li, Guijuan Zhao, Lianshan Wang, Zhen Chen, Shaoyan Yang

**Affiliations:** 1 Key Laboratory of Semiconductor Materials Science and Beijing Key Laboratory of Low Dimensional Semiconductor Materials and Devices, Institute of Semiconductors, Chinese Academy of Sciences, P. O. Box 912, Beijing 100083, China; gjzhao@semi.ac.cn (G.Z.); ls-wang@semi.ac.cn (L.W.); sh-yyang@semi.ac.cn (S.Y.); 2 University of Chinese Academy of Sciences, Beijing 100049, China; 3 LatticePower (Jiangxi) Corporation, No. 699 North Aixihu Road, National High-Tech Industrial Development Zone, Nanchang 330029, China; zchen99@gmail.com

**Keywords:** III-nitrides, nanowires, metal substrates, indium nitride, metal-organic chemical vapor deposition

## Abstract

Growth of semiconductor nanowires on cheap metal substrates could pave the way to the large-scale manufacture of low-cost nanowire-based devices. In this work, we demonstrated that high density InN nanowires can be directly grown on brass substrates by metal-organic chemical vapor deposition. It was found that Zn from the brass substrates is the key factor in the formation of nanowires by restricting the lateral growth of InN. The nanowire morphology is highly dependent on the growth temperature. While at a lower growth temperature, the nanowires and the In droplets have large diameters. At the elevated growth temperature, the lateral sizes of the nanowires and the In droplets are much smaller. Moreover, the nanowire diameter can be controlled in situ by varying the temperature in the growth process. This method is very instructive to the diameter-controlled growth of nanowires of other materials.

## 1. Introduction

In the past several decades, III-nitrides have been successfully applied in various (opto)electronic devices such as light-emitting diodes (LEDs), photodetectors, laser diodes, and high electron mobility transistors because of their remarkable optical and electronic properties [1,2,3,4,5,6,7,8,9,10]. Additionally, III-nitrides are direct bandgap semiconductors and have very wide bandgap range (0.68–6.2 eV) [11,12,13], making them promising candidates for large-scale applications such as photovoltaics (PVs) [14,15,16], photocatalysis [17,18,19], and photocatalytic water splitting [20,21,22]. Moreover, due to the large piezoelectricity of III-nitrides, they were considered as potential materials for flexible (opto)electronic devices [23,24,25]. However, the III-nitrides were conventionally grown on single crystalline substrates (sapphire, Si, SiC, etc.), which are expensive and size-limited, hindering their usage in these large-scale applications. Furthermore, these single crystalline substrates are rigid, and the epilayers must be transferred to other substrates such as polyimide or metals in the fabrication of flexible (opto)electronic devices [26,27,28]. However, due to the high mechanical strength and chemical inertness of III-nitrides, separating them from the substrates is very difficult and time consuming [29,30,31,32].

On the other hand, metal foils are ideal substrates for the large-scale growth of semiconductors because they have large wafer size, low manufacturing cost, and high mechanical flexibility. However, it is usually very difficult to grow single crystalline semiconductor films on metals due to the lack of a global epitaxy. Therefore, semiconductor nanostructures are usually considered as alternative choices. Although there have been many works on the synthesis of Si [33,34], Ge [35,36], and ZnO [37,38] nanowires on various metal substrates, the growth of III-nitrides on metals has rarely been reported [39,40]. Wölz et al. demonstrated that GaN nanowires can be grown on sputtered Ti films by plasma-assisted molecular beam epitaxy (PAMBE) in a self-induced way [39]. The GaN nanowires are well aligned and have a strict epitaxial relationship with the substrate film. Sarwar et al. reported the fabrication of GaN nanowire LEDs on the thin Mo and Ti films on Si wafers by PAMBE [40]. Photoluminescence (PL) measurements indicated that nanowires grown on metal films possess a crystalline quality similar to nanowires grown on single crystalline Si wafers. Recently, high quality GaN nanowires and nanowire-based LEDs have been successfully fabricated on several bulk metal foils, such as Mo, Ti and Ta substrates [41,42]. These results demonstrated that III-nitride nanostructures can be grown on largely scalable substrates that do not have a global epitaxial relationship. However, these works mainly focused on the growth of GaN, and the fabrication of other III-nitrides (InN, AlN) on metal substrates is still lacking.

In this work, we focus on the growth of InN nanowires on metal substrates. Among the III-nitrides, InN has the smallest band gap and effective electron mass [11,12,43], which is attractive for high-frequency electronic devices, near-infrared optoelectronics, and high-efficiency solar cells [44]. Moreover, because of the high intrinsic surface donor density, InN is considered as a very promising material for highly sensitive detection of ions, gases, vapors, and liquids [45,46,47,48]. Moreover, the InN nanowire nanogenerator has the largest output voltage of up to 1 V due to the large piezoelectricity of InN [49,50]. Therefore, the growth of InN on cheap metal substrates is very attractive because it could pave the way for the large-scale manufacture of various low-cost InN-based devices. In our previous work [51], we found that InN nanowires can be grown on amorphous glass substrates. However, an additional dopant source and buffer layers were still needed. Here, we demonstrate that InN nanowires with controllable morphologies can be directly grown on brass (Cu–Zn alloy) substrates via metal-organic chemical vapor deposition (MOCVD) without introducing any foreign catalyst source. The nanowires are obtained via the self-catalyst vapor-liquid-solid (VLS) process with the spontaneous formation of In droplets. The Zn species in the substrate is found to play an important role on the nanowire formation by limiting the lateral growth of InN nanowires [52,53,54]. It was found that the diameters of the nanowires are highly related to growth temperature. The nanowire diameter is typically larger than 100 nm under low growth temperature (440 °C) but is less than 50 nm under higher growth temperature (500 °C). By changing the temperature in the sample growth process, nanowires with varying diameter can be obtained.

## 2. Experimental

The sample growth was performed in a homemade metal-organic chemical vapor deposition (MOCVD) system, which has been described by previous researchers in our group [55]. Brass foils (composed of ~62% Cu and ~38% Zn, ~100 μm thick Yuema, Shanghai, China) were used as the substrates. The furnace was kept at atmospheric pressure in the entire growth process. Prior to growth, the furnace was purged with nitrogen (Air Liquide, Beijing, China) at 6 SLM (standard liter per minute). Then, the furnace was ramped (50 °C/min) from room temperature to the growth temperature (420–520 °C) for the sample growth. Trimethylindium (TMIn, Nata Opto-electronic Material, Suzhou, China) and ammonia (Linggas, Tianjin, China) were used as the In and N precursors, respectively. High-purity nitrogen was used as carrier gas. The flow rates of TMIn and ammonia are 14 μmol/min and 3 SLM, respectively. The nanowire growth process lasted for 5–60 min. Finally, the TMIn flow was cut off and the furnace was cooled down to room temperature under the protection of ammonia flow.

The morphologies and composition of the samples were examined via scanning electron microscopy (SEM: Nova NanoSEM 650, FEI, Hillsboro, OR, USA) and transmission electron microscopy (TEM: JEM 2100F, 200 kV, JEOL, Tokyo, Japan). The crystal structure of the products was characterized by X-ray diffraction (XRD: Philips X’pert Pro X-ray diffractometer, Almelo, Netherlands) with Cu Kα radiation of 0.15406 nm.

## 3. Results and Discussion

### 3.1. Morphology and Structural Characterization

**Figure 1:**
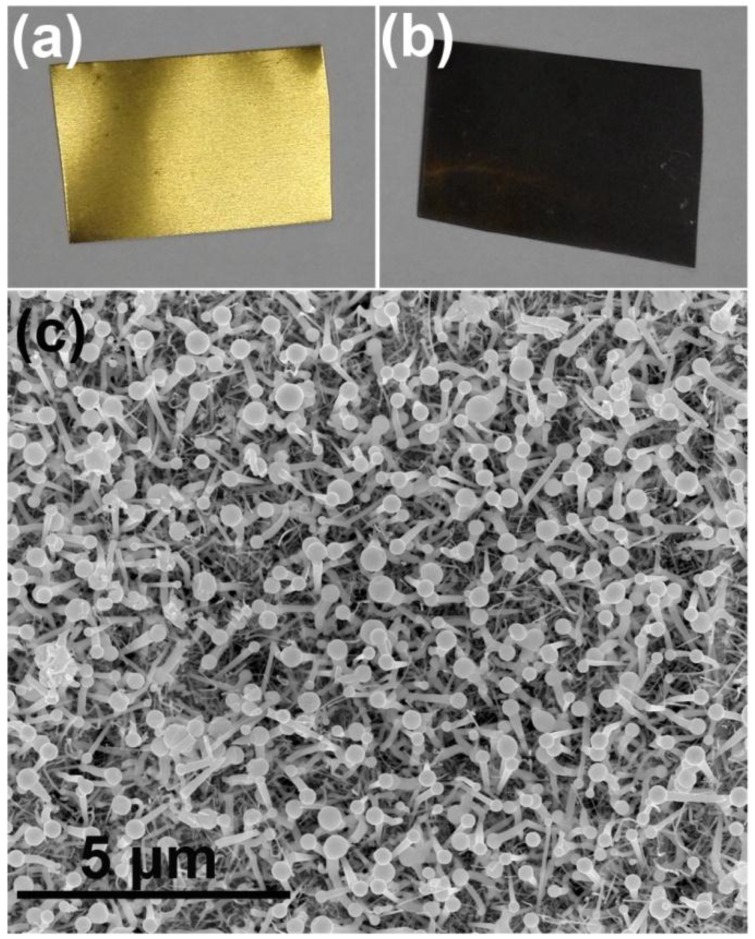
InN nanowires grown on the brass substrates. (**a**,**b**) Digital photographs of the brass substrate before and after nanowire growth. (**c**) Bird view scanning electron microscopy (SEM) image of the InN nanowires.

**Figure 2:**
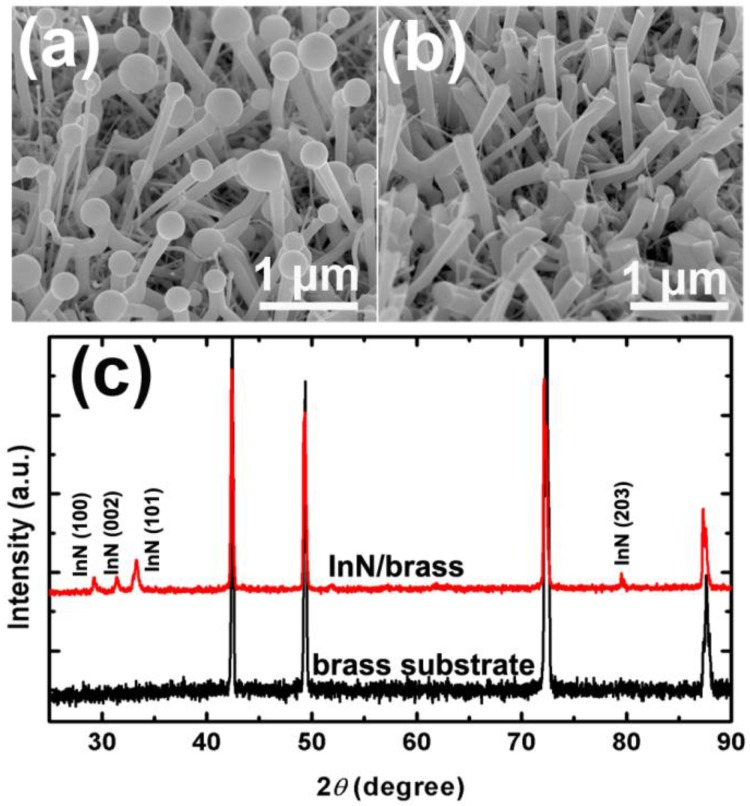
SEM and X-ray diffraction (XRD) characterization of the InN nanowires. Tilted view SEM images of (**a**) the as-grown nanowires and (**b**) the nanowires etched in diluted HCl solution; (**c**) XRD pattern of the nanowires (upper line) and the original brass substrate (bottom line).

After growth of the nanowire for 60 min at 440 °C, the surface of the wafers turned from yellow and mirror-like to black and rough, as shown in Figure 1a,b. The color of the nanowire samples is similar to those grown on sapphire substrates [52,53,54]. This is because the bandgap of InN is small such that almost all the visible light is absorbed by the InN nanowires. The SEM image of the product shows highly dense nanowires (Figure 1c). It can be seen that each nanowire has a droplet on the end, which is strong evidence that the nanowires are grown via the VLS process. The diameter of the nanowire top is a little larger than the bottom (Figure 2a). After being dipped in diluted hydrochloric acid (HCl) solution for about 20 min, the droplets disappeared and the upper surfaces of the nanowires were exposed (Figure 2b). Since no foreign catalyst was used, it is reasonable to infer that these droplets on the nanorods are metal In droplets [52]. Figure 2c shows the XRD spectra of the nanowire sample and the original substrate. Besides the peaks of the brass substrate, diffraction from (101), (002), (102) and (203) planes of wurtzite-type InN was observed in the nanowire sample, suggesting that we have successfully prepared wurtzite InN nanowires on brass substrates.

**Figure 3:**
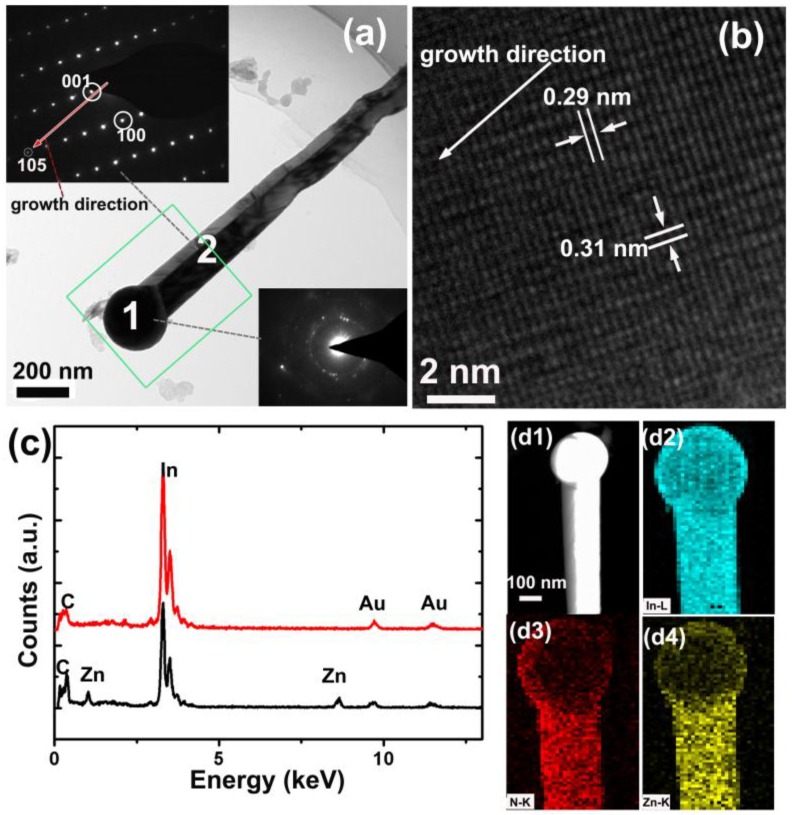
Structural characterization of the InN nanowires. (**a**) Transmission electron microscope (TEM) and (**b**) high-resolution TEM images of a nanowire. The insets in (**a**) are the selected area electron diffraction (SAED) patterns of the In droplet and the nanowire, respectively marked as 1 and 2 of the product; (**c**) Energy-dispersive X-ray spectroscopy (EDXS) spectra of the In droplet (upper line) and the InN nanowire (bottom line). (**d1**) The high-angle annular dark-field (HAADF) image collected from the box labeled area in (**a**). (**d2**–**4**) The EDXS elemental mapping of the box labeled area of the nanowire.

To further analyze the microstructures of the nanowires, TEM and corresponding selected area electron diffraction (SAED) were examined (Figure 3). The length of the nanowire is about 1.5 μm, and the lateral sizes at the bottom and the top are about 100 nm and 160 nm, respectively. The metal droplet at the end of the nanowire is about 250 nm in diameter, which is apparently larger than that of the nanowire. The insets in Figure 3a are the SAED patterns of the droplet and the nanowire, respectively. It can be seen the metal droplet is poly-crystalline while the nanowire is nearly single-crystalline. The crystal planes corresponding to the different diffraction spots are marked in Figure 3a insets, which indicate that the nanowire was grown in the semipolar direction. However, the crystallographic orientation of the nanowires is not unique because other orientations were also observed (Figure S1). Figure 3b is the high-resolution lattice image. The interplanar distances of 0.29 and 0.31 nm match well with the *d*_002_ and *d*_100_ spacing of the wurtzite-type InN. We found that the crystal quality of these products is not as high as those grown on sapphire substrates [52,54,56]. In those works, the In droplets were found to be single crystalline and have an epitaxial relationship with the substrates. In this paper, the In droplets are poly-crystalline, and obvious crystal defects can be observed in some nanowires (Figure S2). Therefore, the lack of a global epitaxial relationship between the nanowires and the substrate may generate defects in the nanowires. However, GaN nanowires with excellent crystal quality were formed on metal substrate by molecular beam epitaxy (MBE). Those GaN nanowires might not have grown via the VLS process because no droplet was found in the products. From these results, we suspect that the non-single-crystalline substrate may not be the only reason of the defect generation in our products. It is possible that the growth method, growth condition, and the substrate combined together to affect the crystal quality of the nanowires. Since the main purpose of this work is to provide a method for growing InN nanowires on metal substrates, the types and origins of these defects will be carefully studied and presented in future works.

To confirm the composition of the metal droplet and the nanowire, energy-dispersive X-ray spectroscopy (EDXS) measurements were carried out as shown in Figure 3c. Au signals are generated from the gold grids that support the nanowires. No Cu signal was found in the droplet or the nanowire, suggesting that the nanowire growth is not affected by the Cu constituent in the substrate, perhaps due to the high melting point of Cu. In the nanowire, Zn signals were found. The Zn/In atom ratio is about 6%. Since no Zn source was intentionally introduced in the growing process, the Zn dopants in the nanowires must come from the substrate. Interestingly, Zn does not exist in the droplet, or there is too little to be detected by EDXS. This phenomenon was also observed in the Zn-doped InN nanorods grown on sapphire substrates [52,54]. The HADDF and the EDXS elemental mapping images of the upper part of the nanowire (box labeled in Figure 3a) are presented in Figure 3d1–4). We can see that the Zn dopant is uniformly distributed in the nanowire. No aggregate of the dopant is observed. The EDXS line scans along the growth direction of and across the nanowire also indicate the Zn dopant is uniformly distributed in the nanowire (Figure S3).

**Figure 4:**
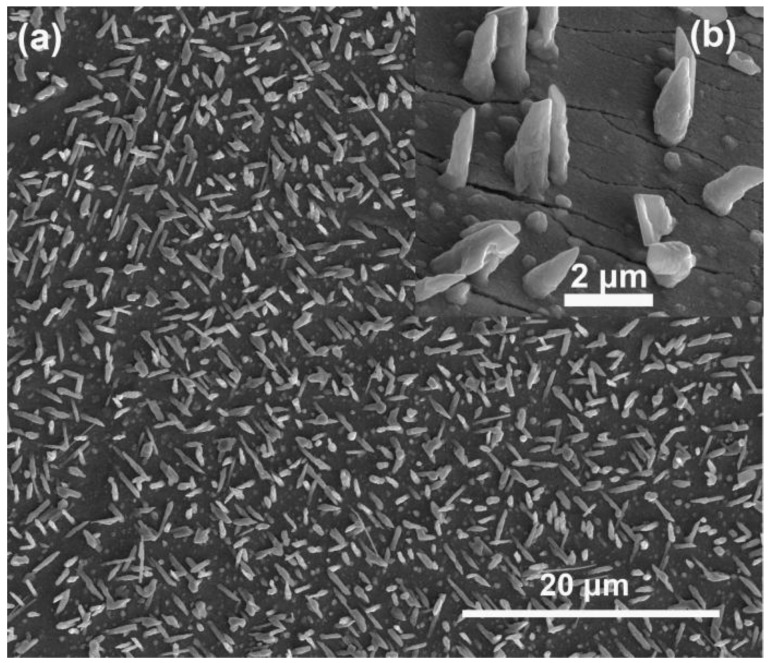
(**a**) SEM image of the InN grown on pure Cu substrates; (**b**) Enlarged SEM image of the InN/Cu products.

In previous literature [52,53,54], Zn doping was considered a key factor in the formation of InN nanorods by limiting the lateral growth of InN. The nanorods could not be grown without Zn even when the In droplet existed. To verify whether Zn plays the same role in this work, we grew InN on the pure Cu substrates (99.999%, Yuema, Shanghai, China). As shown in Figure 4, the InN grown on Cu substrates has a cone-like morphology. The lateral size at the bottom of the nano-cones is typically about 500 nm, which is obviously larger than those grown on the brass substrates. Thus, we can infer that, without Zn, the lateral growth of InN would not be restricted, and no nanowire can be obtained. Moreover, no In droplet was found at the end of the nano-cone. This also indicates that the Zn plays a crucial role in the formation of the In droplets, which is consistent with the observation by Zhang et al. [54]. The growth mechanisms of InN nanowires on metal substrates is presented in Section 3.3.

### 3.2. Effect of the Growth Temperature

**Figure 5:**
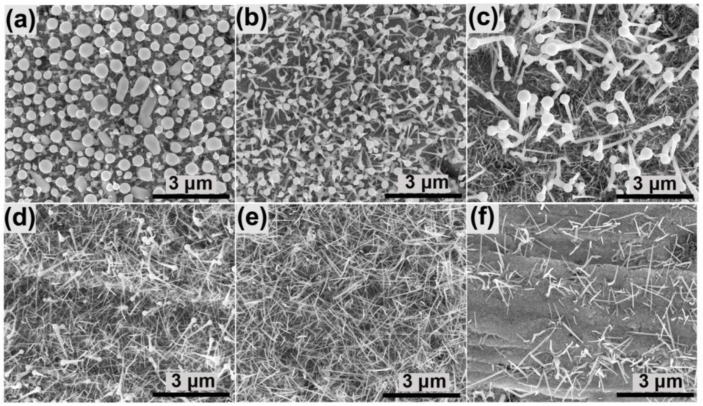
SEM images of the nanowires grown at (**a**) 420 °C; (**b**) 440 °C; (**c**) 460 °C; (**d**) 480 °C; (**e**) 500 °C and (**f**) 520 °C.

Figure 5 shows the nanowire morphologies grown for 40 min under different growth temperatures (420–520 °C). It is obvious that the nanowire morphology is highly dependent on growth temperature. At a low growth temperature (420 °C), the In droplets at the end of the nanowires are about several hundred nanometers in size (Figure 5a), which is significantly larger than those grown at higher temperatures. The reason for this might be that the decomposition of ammonia is slow at a low temperature and the N source is insufficient compared to the In source. The excess In source was incorporated into the In droplets and thus increased in droplet size. At 440 °C (Figure 5b), the nanowires are uniformly distributed with a diameter of about 100 nm. Under a higher growth temperature (460 °C), the product is composed of two kinds of nanowires (Figure 5c). One kind of nanowire has a larger diameter (~100 nm, thick nanowires), while the other exhibits a much smaller diameter (less than 50 nm, thin nanowires). Different from the thick nanowires, no obvious lateral size difference between the droplets and wires was found on the thin nanowires. The amount of the thick nanowires decreases at 480 °C (Figure 5d), and almost no thick nanowire exists in the sample grown at 500 °C (Figure 5e). A further increase in the growth temperature did not result in highly dense nanowires (Figure 5f), perhaps due to the high decomposition rate of InN at high temperatures.

**Figure 6:**
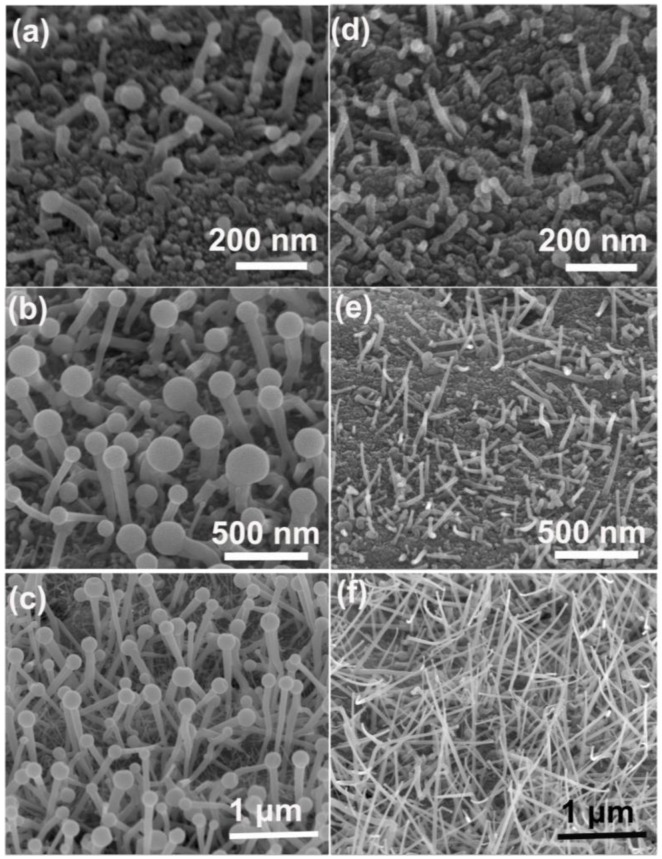
SEM images of the nanowires grown for different time. (**a**–**c**) The nanowires grown at 440 °C for 5, 20 and 40 min, respectively; (**d**–**f**) The nanowires grown at 500 °C for 5, 20 and 40 min, respectively.

To seek the reason of the morphology difference between the nanowires grown at low and high temperatures, two sets of nanowire samples grown for different lengths of time were prepared. Figure 6a–c show the nanowires grown at 440 °C for 5, 20 and 40 min, respectively. It can be seen that the nanowires and the In droplets have a small size at the initial growth stage (Figure 6a). As the growth time increases, the size of the In droplets increases rapidly. The diameter of the nanowires is also increased, although not as fast as the In droplets. For comparison, the diameter of the nanowires and droplets grown at a high temperature (500 °C) does not increase much at the different growth time (Figure 6d–f). This might be due to the fact that, at a high growth temperature, the reaction rate of In and N sources is fast such that no excess In vapors are incorporated by the In droplets. The diameter of the nanowires is limited by the In droplet and thus cannot increase apparently. At moderate temperatures (460–480 °C), only some of the In droplets can grow larger; therefore, the products are composed of two kinds of nanowires.

### 3.3. Growth Mechanisms of the Nanowires

**Figure 7:**
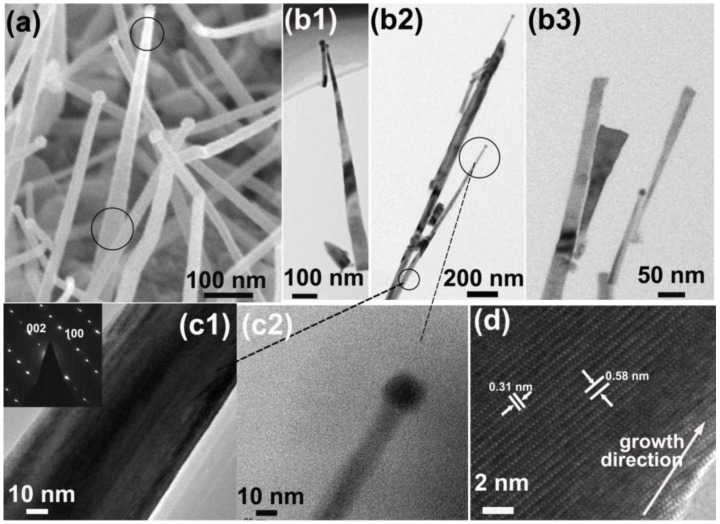
(**a**) Enlarged SEM image of the nanowires with small lateral size. (**b1**–**3**) TEM images of these nanowires. (**c1**–**2**) Enlarged TEM images of the marked places of a nanowire in Figure 2b. The inset in (**c1**) is the SAED pattern. (**d**) High-resolution TEM image of the nanowire.

To elucidate the growth mechanism of the InN nanowires on metal substrates, we present here more characterizations of the thin nanowires and the statistical analysis of the nanowire size at different growth conditions. Figure 7a shows an enlarged SEM image of the thin nanowires. The top and bottom of the nanowires were marked with circles. We can see that the bottom lateral size is larger than the top lateral size, which is opposite to the thick nanowires (see Figure 2 and Figure S4). The TEM images in Figure 7b1–3,c1–2 also confirm this. This phenomenon implies the growth mechanisms are different between the small and larger nanowires. SAED pattern (Figure 7c1 inset) and high resolution TEM image (Figure 7d) of the nanowire indicate the nanowire was also grown in the semipolar direction.

**Figure 8:**
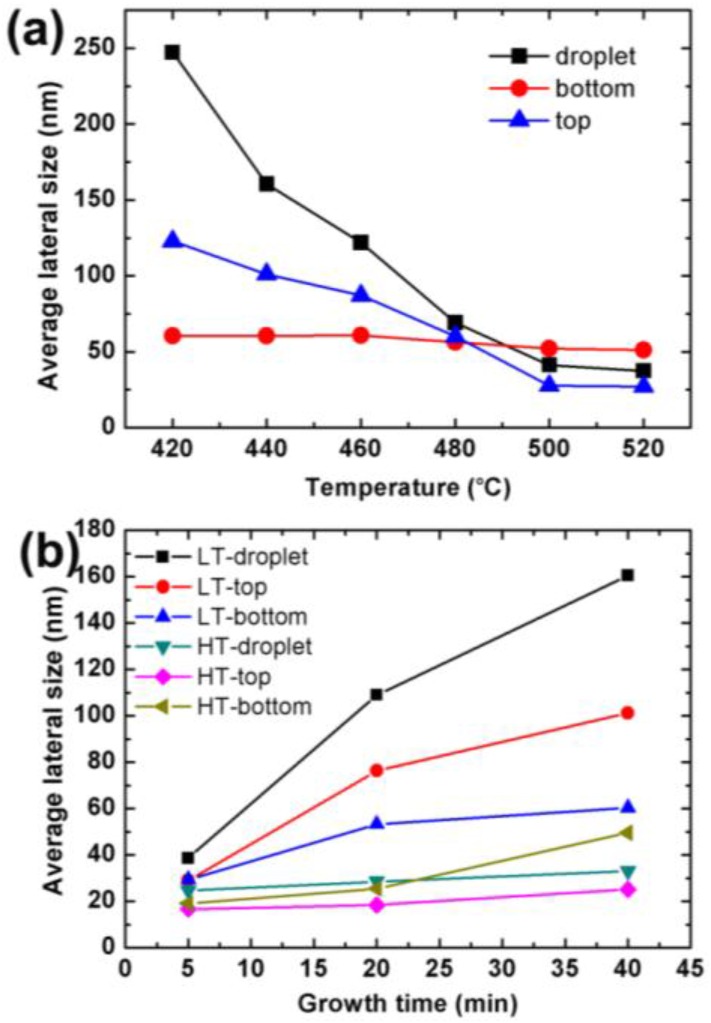
The statistical average lateral size of the droplets, the bottom, and the top parts of the nanowires at different growth conditions. (**a**) The lateral size of the droplets and nanowires grown at different temperatures; (**b**) The lateral size of the droplets and nanowires grown for different time at a low temperature (LT, 440 °C) and a high temperature (HT, 500 °C), respectively.

The average diameters of the In droplets, the bottom, and the top parts of the nanowires grown at different temperatures and stages were obtained by calculating the mean value of more than 100 nanowires at each growth condition. Figure 8a shows the average lateral size of the nanowires and droplets grown at 420–520 °C for 40 min. We can see that the lateral size of both the droplets and the nanowires drops with the growth temperature. The size of the droplets drops more rapidly than both the top and the bottom size of the nanowires. Interestingly, the bottom size of the nanowires almost remains unchanged with the temperature, while the top size of the nanowires apparently decreases. For all growth temperatures, the top size of the nanowires is smaller than that of the droplets. In contrast, the bottom size of the nanowires grown above 500 °C becomes larger than the droplet size.

**Figure 9:**
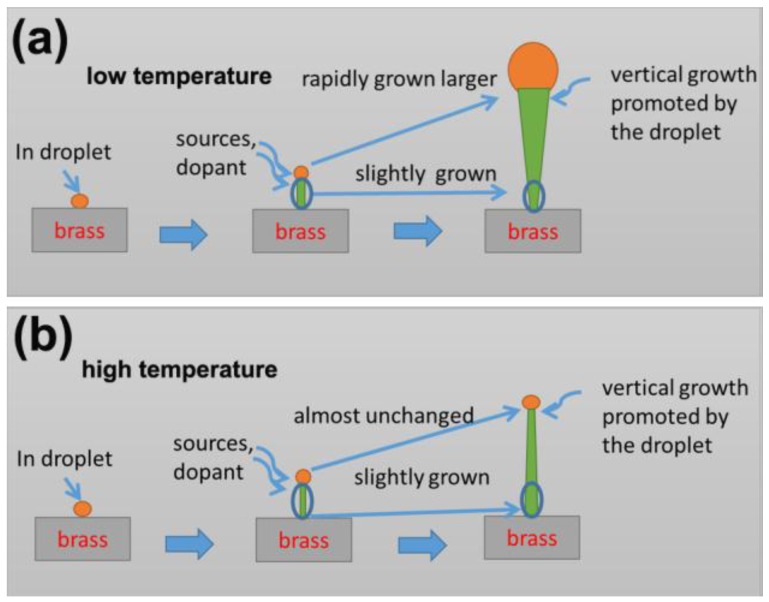
Schematic illustrations of the growth mechanisms of the InN nanowires grown at (**a**) low and (**b**) high temperatures.

The lateral size of the nanowires grown for 5–40 min at 440 °C and 500 °C is shown in Figure 8b. We can see that the droplets and the top of the nanowires grow larger rapidly at a low temperature. It might be due to the insufficient reaction between In and N source at low temperatures, stated in Section 3.2. It can also be seen that the bottom size of the nanowires changes very slowly, which indicates that the lateral growth of the nanowires was limited. In a previous work by Song et al. [53], it was found that, while the Zn dopant source was cut off in the InN nanowire growth process, InN started to grow rapidly on the nanowire sidewalls. They inferred that the InN nanowire lateral size was limited by the presence of Zn dopants. In this work, we found that the bottom size of the nanowires changes slowly when the Zn dopants exist and is obvious smaller than the products grown without Zn dopants (Figure 4). Therefore, we can infer that the lateral growth of the InN nanowires on brass substrates could also be limited by the existence of Zn dopants. Although the top size of the nanowires grows rapidly, it is more likely to be caused by the vertical growth of the nanowires underneath the droplets. In the VLS growth process of nanowires, the vapor-phase source species diffused through the droplets and supersaturated at the liquid/solid interface to produce nanowires [57]. For the low temperature-grown samples in this work, since the In droplets increase rapidly, the top part of the nanowires grown underneath the droplets should also increase. At a high growth temperature, the diameters of the In droplets and the InN nanowire top almost remain unchanged with the growth time. This might be due to the enhanced InN growth and reduced droplet growth at high growth temperatures. The bottom size grows slightly larger at a longer growth time and becomes larger than the droplets at 40 min. This indicates that the lateral growth of the nanowires was not totally inhibited. Since the nanowire bottom has been exposed to the growth ambient for longer time than the nanowire top, the nanowires grown at a high temperature have a larger lateral size at the bottom, which can be seen in Figure 7. The growth mechanisms of the InN nanowires on brass substrate at low and high temperatures are schematically illustrated in Figure 9 for a clear view.

### 3.4. Growth of Multi-Section Nanowires

**Figure 10:**
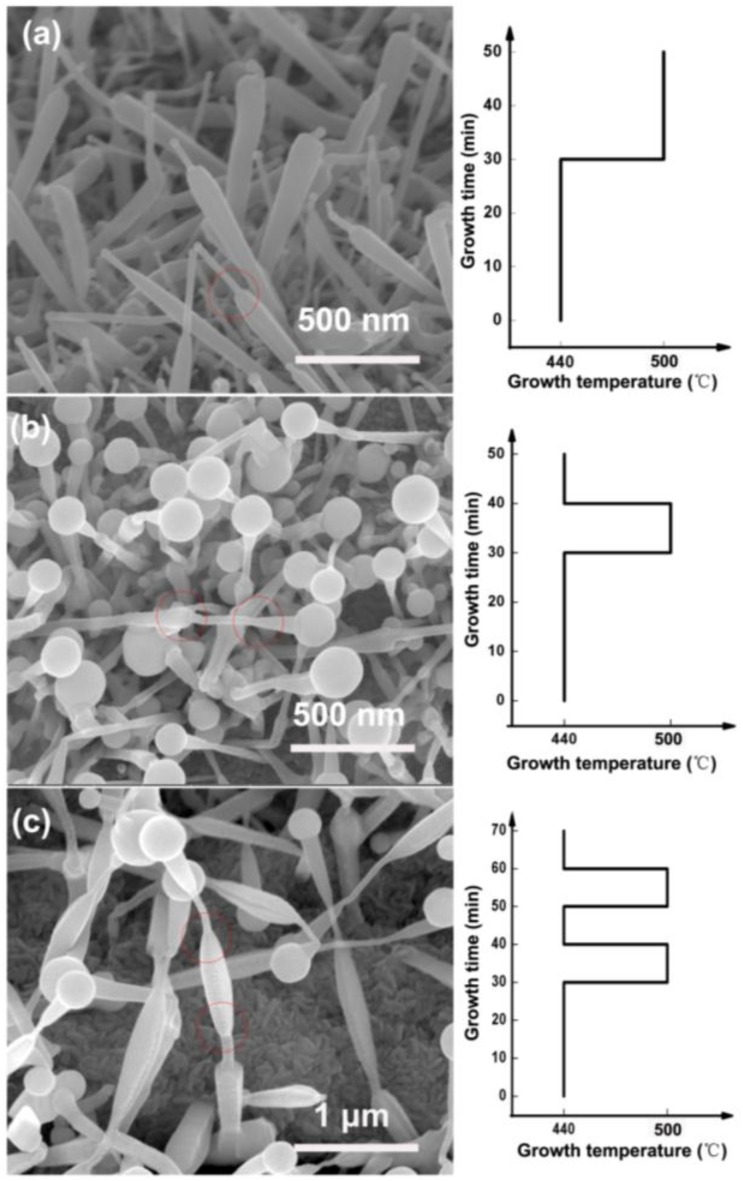
The nanowires formed in situ via temperature variation during the growth process. The growth procedures were illustrated at the right side of each SEM image. **(a**) The nanowires grown at 440 °C for 30 min and then grown at 500 °C for 20 min; (**b**) The nanowires grown at 440 °C for another 10 min after the low-to-high temperature growth process; (**c**) The nanowires grown with an additional temperature variation step.

Since the diameter of the nanowires is highly dependent on the growth temperature, it is reasonable to infer that multi-section nanowires can be obtained by varying the temperature in the growth process. Figure 10a shows the nanowires grown at 440 °C for 30 min and then grown at 500 °C for 20 min. The diameter of the nanowires is large at the lower part and much smaller at the upper part. At the marked place in Figure 10a, we can clearly see the diameter variation. The size of the In droplets of these nanowires are similar to those grown at 500 °C (Figure 5e). The morphology of these nanowires further proves that the size of the In droplets decreases at higher temperatures. The lateral size of the nanowires is limited by the In droplets and thus can be controlled in situ via temperature variation. In Figure 10b, the nanowires were grown at 440 °C for another 10 min after the low-to-high temperature growth process. It can be seen that the diameter of the nanowires was first increased and then decreased, as shown in the figure. The In droplets were changed to large spheres due to the additional low temperature growth process. Figure 10c shows the nanowires grown with an additional temperature variation step, which leads to two segment pairs of “wide-narrow-wide” diameter configurations. In previous works, researchers found that the diameters of ZnO or GaN can be changed by controlling the source supply [37,58]. In this work, a new method to control the nanowire diameter is demonstrated, which is instructive with respect to the diameter-controlled growing nanowires of other materials.

## 4. Conclusions

In conclusion, InN nanowires were successfully grown on brass substrates via MOCVD. High-density nanowires with In droplets on the end were formed. It was found that Zn played an important role in the formation of nanowires by limiting the lateral growth of InN. The nanowire morphology is highly dependent on the growth temperature. While at a lower growth temperature, the nanowires have a large diameter and In droplets. At the elevated growth temperature, the lateral sizes of the nanowires and the In droplets are much smaller. The growth mechanisms of the nanowires on brass substrates at different temperatures were presented. By varying the temperature in the growth process, we found that the nanowire diameter can be controlled in situ. The successful preparation of InN nanowires on cheap metal substrates could pave the way for the large-scale manufacture of low-cost InN-based devices. Moreover, the in situ control of the InN nanowire diameter by varying the growth temperature is instructive to the diameter-controlled growing nanowires of other materials.

## Supplementary Materials

The following are available online at http://www.mdpi.com/2079-4991/6/11/195/s1.
