# Supplementary material for: Morphology Controlled Fabrication of InN Nanowires on Brass Substrates
Source: Nanomaterials (Basel). 2016 Oct 29;6(11):195. doi: 10.3390/nano6110195 (PMC5245764; doi:10.3390/nano6110195)
Supplement: Supplementary file 1 [file nanomaterials-06-00195-s001.pdf]

Supplementary Materials: Morphology Controlled Fabrication of InN Nanowires on Brass Substrates

Huijie Li, Guijuan Zhao, Lianshan Wang, Zhen Chen and Shaoyan Yang

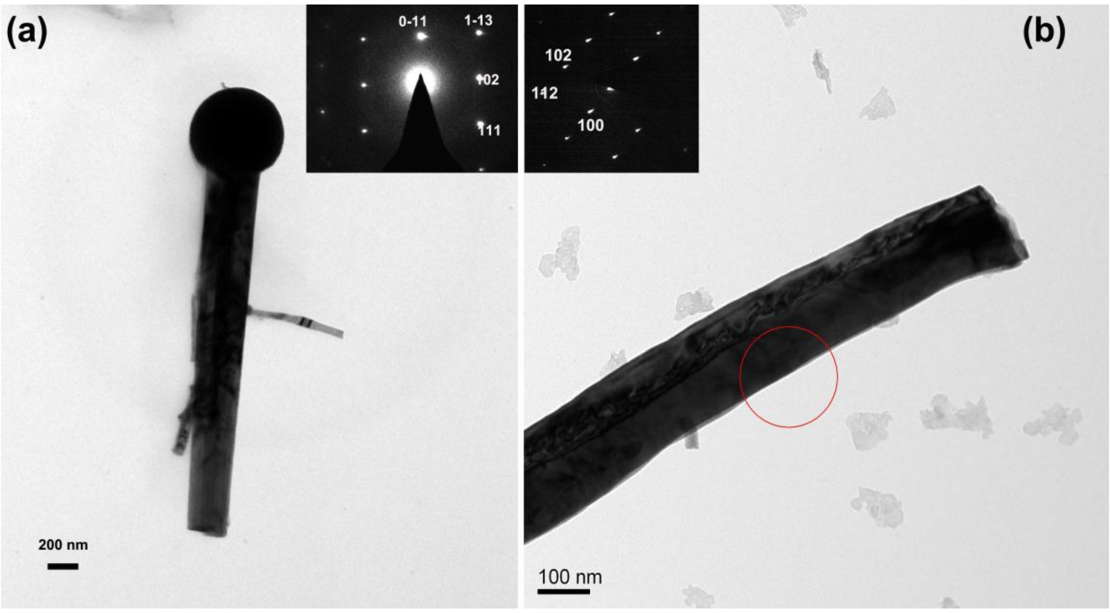

Figure S1. (**a**,**b**) Transmission electron microscopy (TEM) images of two different InN nanowires. Insets are the selected area electron diffraction (SAED) patterns of the nanowires, which indicate the nanowires are grown along different orientations.

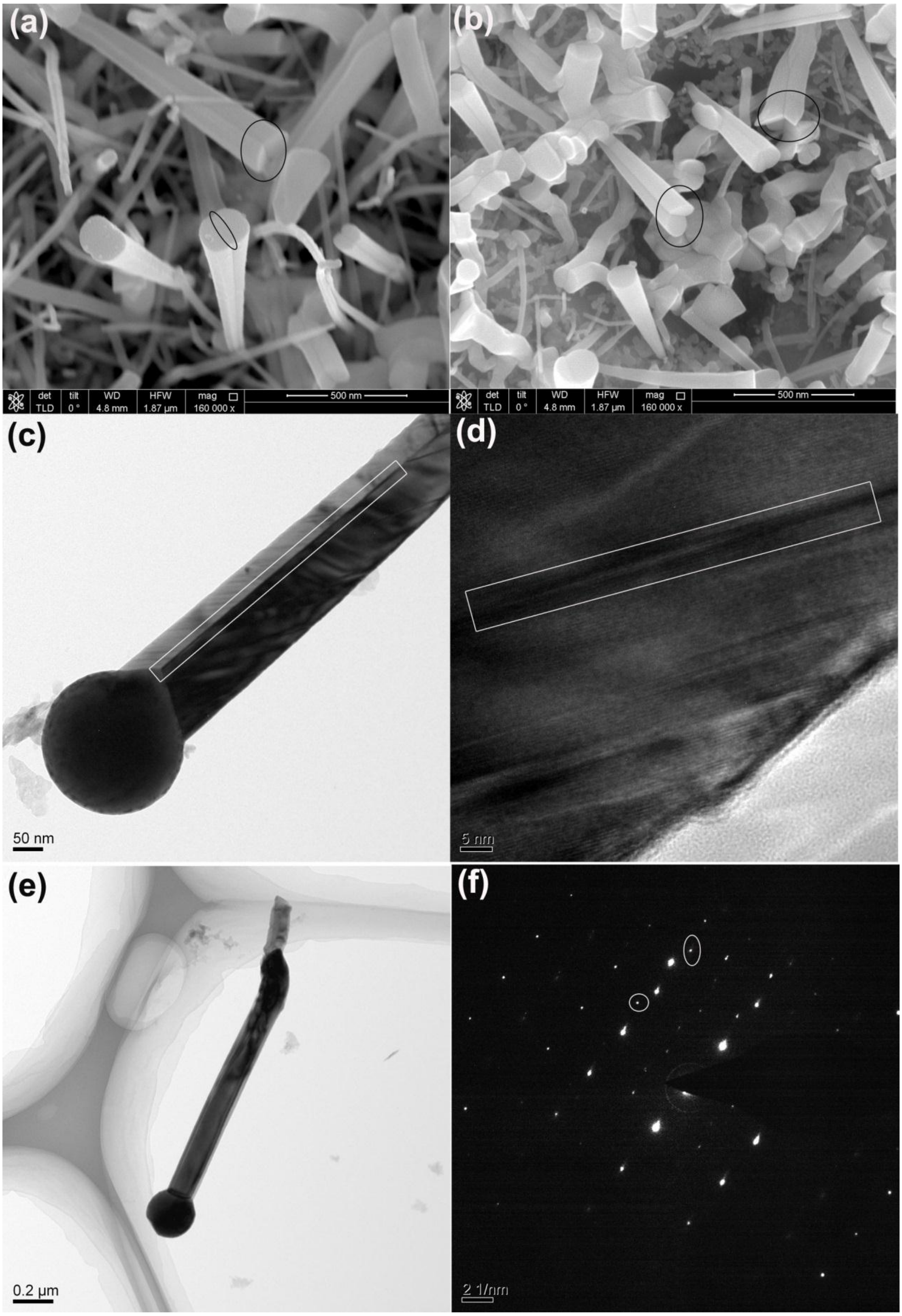

Figure S2. (**a**,**b**) Scanning electron microscopy (SEM) images of the HCl-etched InN nanowires. Clear defects which may be related to the grain boundaries were found at the marked places. (**c**,**d**) TEM images of the nanowires, which proves the existence of some defects. (**e**,**f**) TEM image and the SAED pattern of a nanowire. Irregular spots were found in the SAED pattern, suggesting the crystal quality of the nanowire is not perfect.

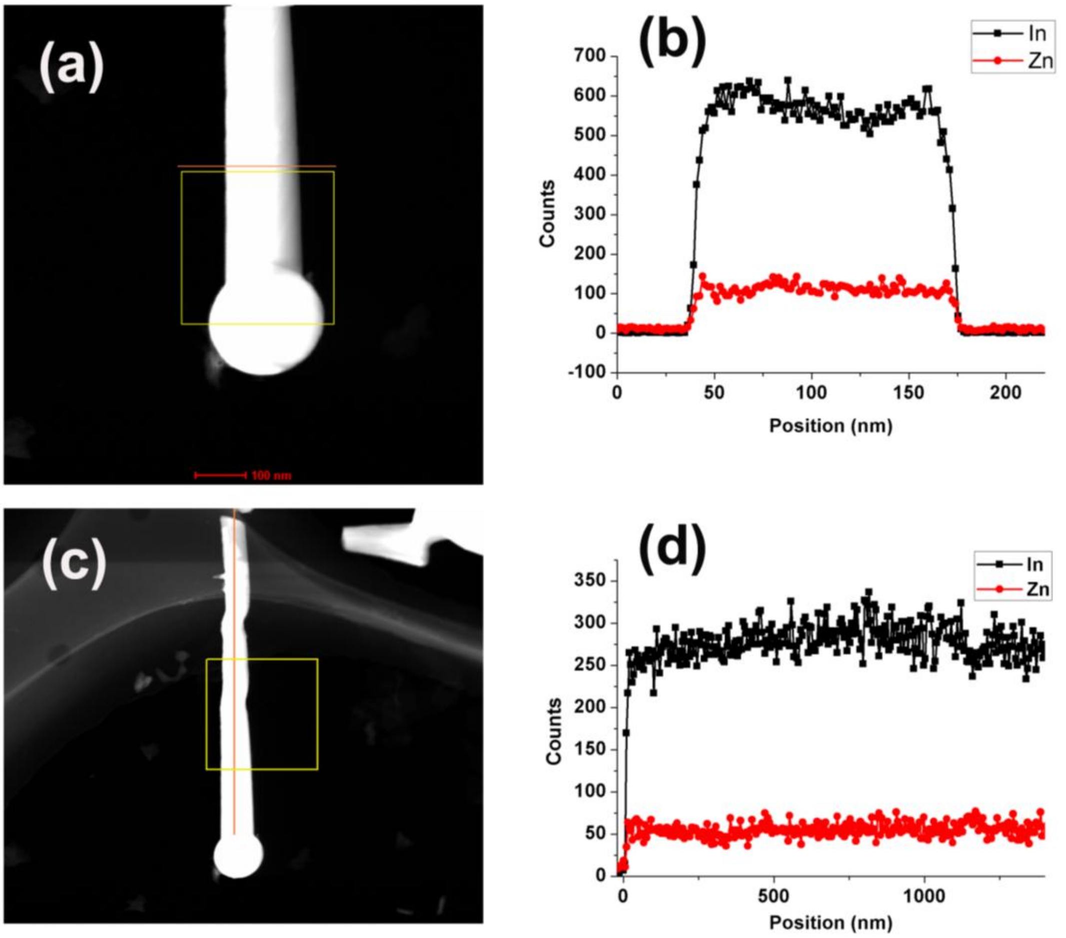

Figure S3. (**a**,**b**) Energy-dispersive X-ray spectroscopy (EDXS) line scan across the nanowire. (**c**,**d**) EDXS line scan along the nanowire growth direction. It can be seen that the Zn profiles are very similar to the In profiles, indicating a homogeneous dopant distribution in the nanowire.

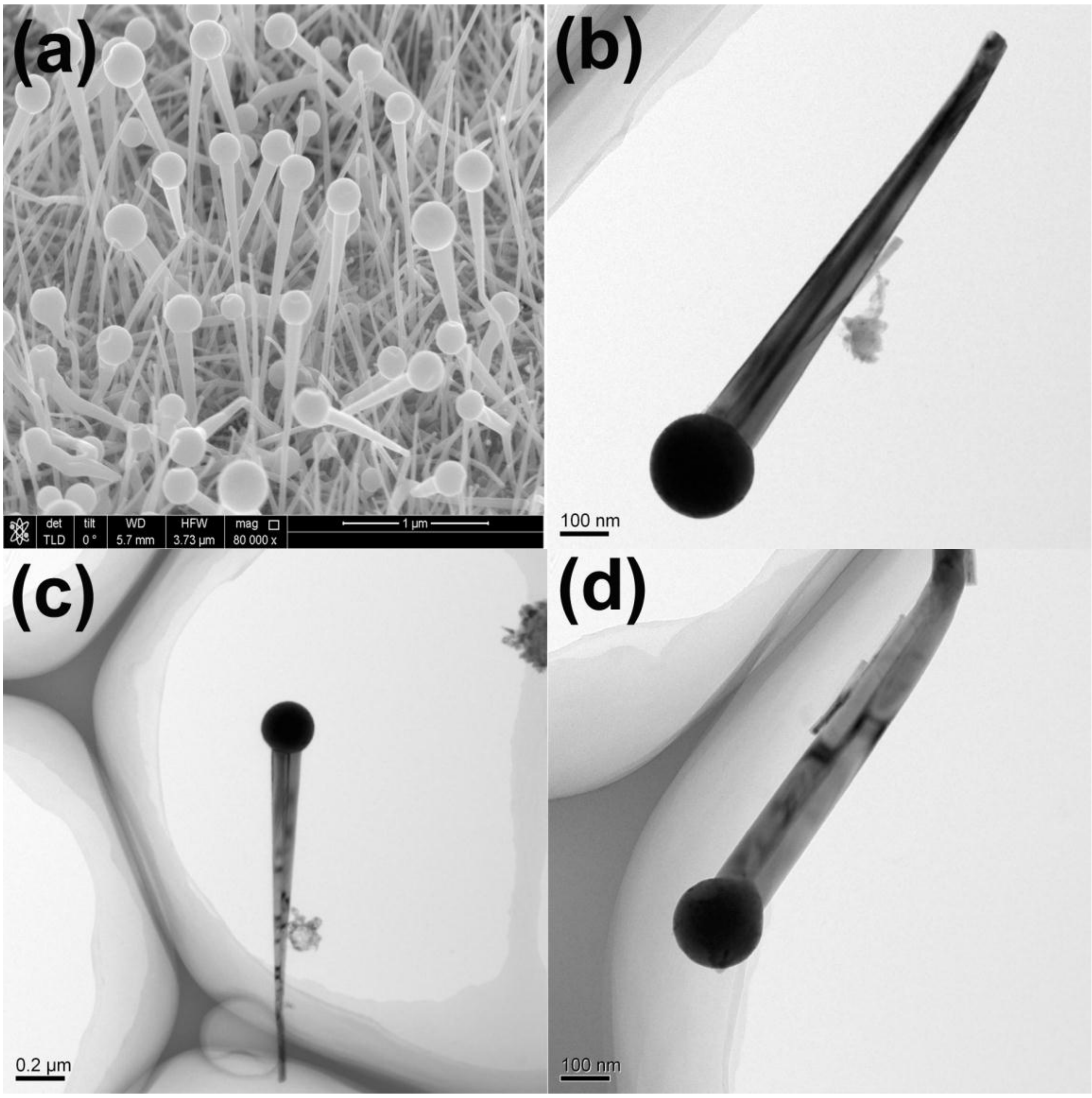

Figure S4. (**a**) SEM and (**b**–**d**) TEM images of the nanowires with larger diameters. It can be seen that the top size is apparently larger than the bottom size of the nanowires.

© 2016 by the authors. Submitted for possible open access publication under the terms and conditions of the Creative Commons Attribution (CC-BY) license (http://creativecommons.org/licenses/by/4.0/).
